# Direct Enantiomeric Resolution of Seventeen Racemic 1,4-Dihydropyridine-Based Hexahydroquinoline Derivatives by HPLC

**DOI:** 10.3390/ijms20102513

**Published:** 2019-05-22

**Authors:** Jiayi Sun, Miyase Gözde Gündüz, Junyuan Zhang, Jia Yu, Xingjie Guo

**Affiliations:** 1 Shenyang Pharmaceutical University, Shenyang 110016, China; 15204077560@163.com (J.S.); zhangjunyuan1995@163.com (J.Z.); 2 Department of Pharmaceutical Chemistry, Faculty of Pharmacy, Hacettepe University, Sıhhiye, Ankara 06100, Turkey; miyasegunduz@yahoo.com or miyaseg@hacettepe.edu.tr

**Keywords:** 1,4-dihydropyridine, hexahydroquinoline, enantioseparation, Chiralpak IC column, high-performance liquid chromatography

## Abstract

1,4-Dihydropyridine (DHP) scaffold holds an outstanding position with its versatile pharmacological properties among all heterocyclic compounds. Although most of the commercially available DHPs are marketed as a racemic mixture, the chiral center at C-4 can lead to even opposite pharmacological activities between the enantiomers. In the present study, enantioseparation of seventeen DHP structural analogues, consisting of either pharmacologically active or newly synthesized derivatives, (M2-4, MD5, HM2, HM10, CE5, N11, N10, N7, M11, MC6-8, MC13, MD23, and 42IIP) by high-performance liquid chromatography was investigated using immobilized polysaccharide-based chiral stationary phase, Chiralpak IC column. Due to the solvent versatility of the covalently immobilized chiral stationary phase in enantiomer separation, multiple elution modes including standard normal phase, nonstandard mobile phase, and reversed phase were used to expand the possibility to find the optimum enantioselective conditions for the tested analytes. Under appropriate separation conditions, complete enantiomeric separation was obtained for nearly all compounds except MC6-8 and MC13 which contained two chiral centers. Additionally, the effects of the polar modifier, the additive, and column temperature on the chiral recognition were evaluated. The thermodynamic parameters calculated according to the linear van’t Hoff equation indicated that the chiral separations in this study were enthalpy-driven or entropy-driven. Some parameters of method validation such as linearity, limit of quantitation, and repeatability were also measured for all studied compounds to prove the reliability of the method.

## 1. Introduction

1,4-Dihydropyridine (DHP) nucleus is one of the most important heterocycles due to its wide variety of biological activities. DHPs, represented by nifedipine, primarily target a specific class of voltage-gated calcium channel, L-type calcium channel (Cav 1.2) in the cardiovascular system, and are used for the treatment of hypertension and angina [1]. Ongoing efforts to explore diverse structural changes on DHP ring yielded a novel class of compounds with condensed heterocyclic core (hexahydroquinoline) possessing various pharmacological properties such as antitubercular, analgesic, and antimalarial, in addition to well-known calcium channel blocking activity [2].

Our group has designed, synthesized, and evaluated the biological profile of numerous DHPs. Among them, some derivatives were identified as effective blockers of different types of calcium channels. While MD5 was completely selective for L-type over T-type [3], M2-4 was found to block both calcium channels effectively [4]. Additionally, N10 was a selective T-type Cav3.2 blocker with significant analgesic effects [5]. Although these compounds were tested as racemates in their original cited works, the enantiomeric resolution is of utmost importance for DHPs because the chiral center at C-4 can lead to even opposite pharmacological activities between the enantiomers [6].

Exploring a stable and reliable method for chiral separation still remains one of the greatest challenges in the last decades for pharmaceutical analysis, especially in the development of new chiral drugs. Many separation technologies such as capillary electrophoresis (CE) [7,8], supercritical fluid chromatography (SFC) [9], capillary electrochromatography (CEC) [10], gas chromatography (GC) [11], and high-performance liquid chromatography (HPLC) [12,13,14,15] have been developed for obtaining efficient chiral separation for DHP enantiomers. Among all these separation techniques, HPLC remains predominant in the analytical scale enantioseparation arena [16]. Moreover, HPLC using chiral stationary phases (CSPs) is the most widely used technique for the direct analysis of enantiomers [17].

The most commonly used CSPs for chiral separations have long been the polysaccharide-based CSPs [18]. Many enantiomers of DHPs have been separated on this kind of CSPs, for example, a study on enantioseparation of benidipine by HPLC has been reported using a hand-made polysaccharides type of CSP [14]. More recently, SFC was also utilized for the separation of seven DHPs on an immobilized polysaccharide-based CSP [9].

**Figure 1:**
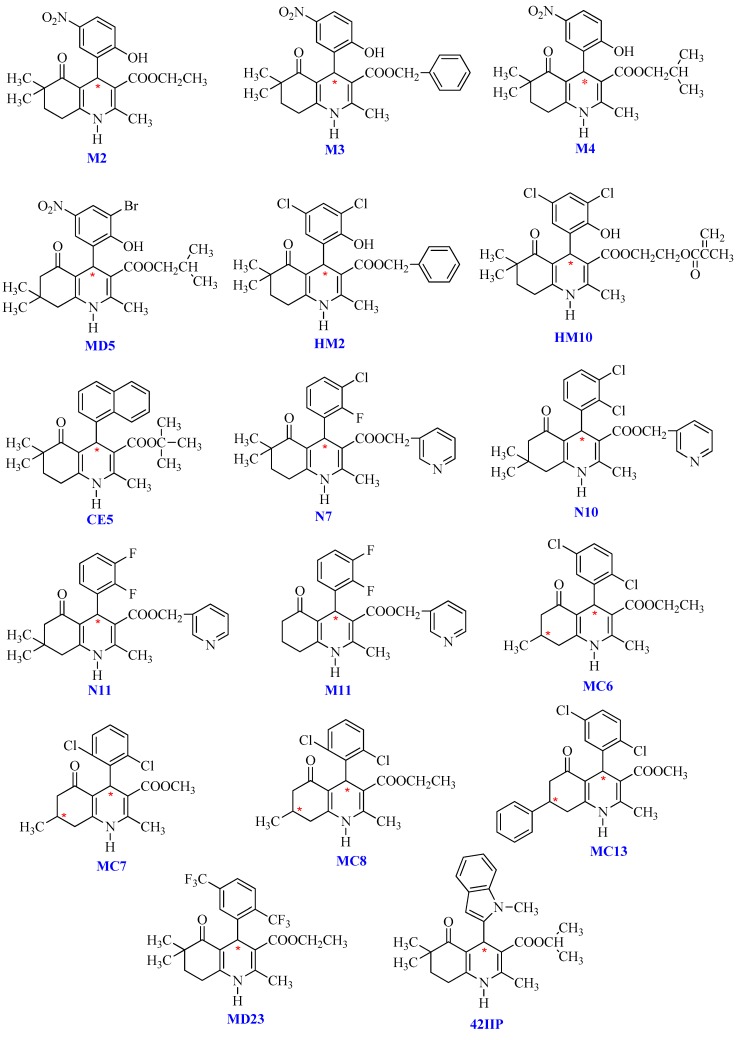
Chemical structures of studied compounds.

Although some studies on applications of the immobilized CSPs for chiral separation of DHPs have been reported [12,13,14,15], the enantiomeric resolution of DHP based hexahydroquinolines, which provide promising scaffolds for the development of novel calcium channel blockers, has not been investigated yet. Meanwhile, among the reported methods, only a few of them have demonstrated the wide solvent versatility of polysaccharide CSPs in enantioseparation of DHPs. These CSPs deserve more attention to enhance our comprehension of their chiral recognition mechanism due to their immobilized characteristic. In the present work, direct HPLC separation of seventeen chiral DHP structural analogues (Figure 1) was investigated on cellulose-based tris (3,5-dichlorophenylcarbamate) Chiralpak IC column under normal-phase, nonstandard, and reversed-phase eluent system. Retention behaviors, effects of the mobile-phase composition, additive concentration and column temperature were systematically studied. Complete resolution was obtained for thirteen tested DHP compounds under its optimized chromatographic condition.

## 2. Results and Discussion

The initial separations of seventeen racemic DHP-based hexahydroquinoline derivatives were investigated on three chiral columns including Chiralcel OD-RH, Chiralpak ID, and Chiralpak IC column. It turned out to be that Chiralpak IC had much better enantioseparation ability for the tested compounds than that of Chiralcel OD-RH and Chiralpak ID. When a Chiralcel OD-RH column was utilized, a reversed mobile phase was used, and only one analyte achieved baseline separation. Moreover, the solvent tolerable for this coated chiral stationary phase is limited, which is not conducive to investigating the influence of various solvents. In the meanwhile, two tested compounds were completely separated on the Chiralpak ID column in normal-phase mode. The chiral separation ability was better than that of the Chiralcel OD-RH column, but still not suitable for the separation of the analytes. Therefore, the Chiralpak IC column was selected for further investigation.

### 2.1. Enantioseparation under Normal-Phase Elution Mode

#### 2.1.1. Under Standard Normal-Phase Elution Mode

**Figure 2:**
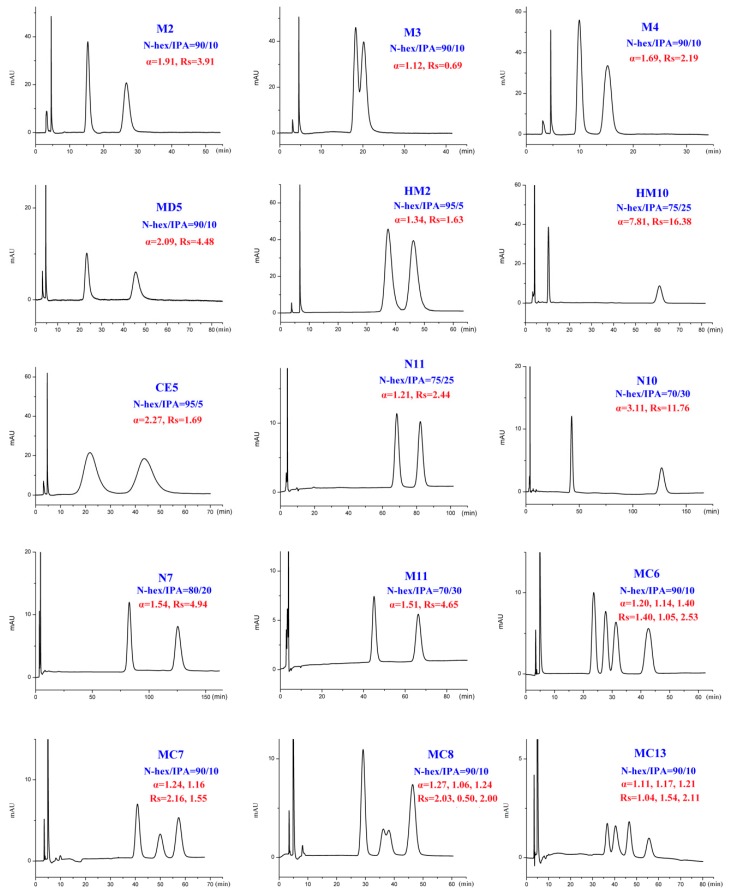
Typical chromatograms with isopropanol (IPA) as organic modifier under normal-phase mode; enantioselectivity factor (α) and resolution (R_s_).

**Figure 3:**
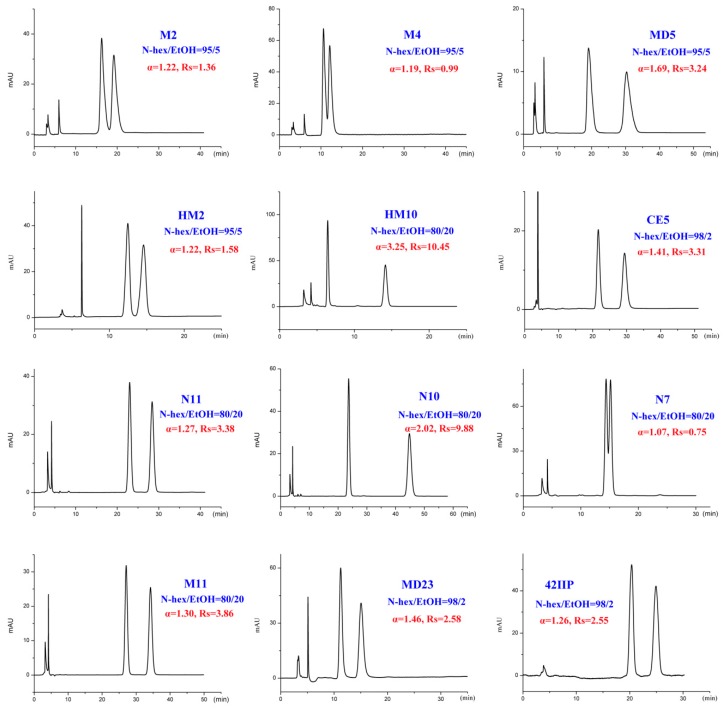
Typical chromatograms with ethanol (EtOH) as organic modifier under normal-phase mode.

**Figure 4:**
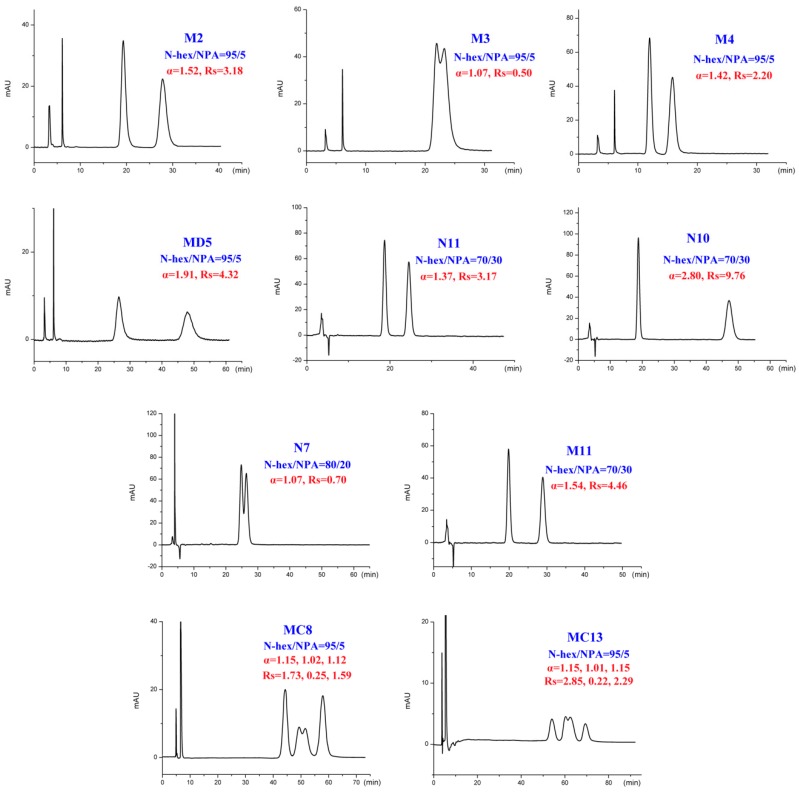
Typical chromatograms with n-propanol (NPA) as organic modifier under normal-phase mode.

**Figure 5:**
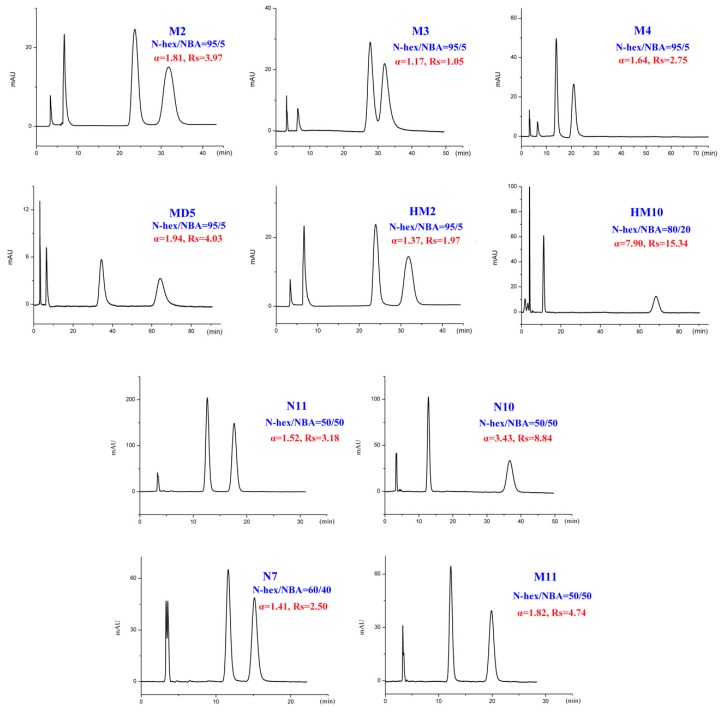
Typical chromatograms with n-butanol (NBA) as organic modifier under normal-phase mode.

The binary mixture of alkanes and various alcohol modifiers are the most commonly applied mobile-phase systems owing to their versatility in providing enantiomeric separations on polysaccharide-based CSPs. During our study, enantiomeric separation of seventeen tested DHP enantiomers (Figure 1) on Chiralpak IC column was primarily performed by normal-phase HPLC consisting of N-hexane (N-hex)/alcohol (isopropanol (IPA), ethanol (EtOH), n-butanol (NBA), and n-propanol (NPA)) in appropriate proportions. It was found that the CSP we selected was enantioselective for the whole set of compounds. Especially, twelve out of the seventeen analytes could be fully resolved under such mobile-phase conditions with no extensive method optimization. In fact, much more screening mobile-phase compositions were applied for the present study and the large number of data can hardly be presented in this paper. To facilitate the discussion, Tables S1–S4 show the partial enantioseparation results after optimization of the nature and concentration of modifiers in the mobile phase. The typical chromatograms associated with tables above are shown in Figure 2, Figure 3, Figure 4 and Figure 5.

In consideration of the results obtained with the four modifiers, it was evident that the application of different modifiers produced significant variation on the resolution on Chiralpak IC. As can be seen by comparing the results in the above tables, the superiority of IPA and NBA over the other two alcohols was apparent. In particular, baseline separation was obtained for ten analytes with IPA or NBA and eight of them (M2-4, MD5, MC6-8, and MC13) achieved maximum resolution with different proportions of IPA as modifier. For instance, the best separation of MD5 (R_s_ = 5.77) was obtained using 5% IPA as modifier. In contrast, the separation profiles of NPA and EtOH was relatively poor on this CSP, as it allowed the baseline separation for nine analytes. Take N7 as an example, the baseline separation was lost upon changing the modifier from IPA/NBA to NPA/EtOH. However, the opposite effect was observed in cases of CE5. In the chiral separation of CE5, EtOH (R_s_ = 3.31) gave better result than NBA (R_s_ = 0).

#### 2.1.2. Under Nonstandard Elution Mode

As demonstrated in previous studies [16], nonstandard mobile-phase systems have many benefits. For instance, different selectivity profile may be achieved owing to the presence of solvents with diverse nature and properties. This helps for the choice of the best method for the resolution of a given pair of enantiomers. Thus, to further widen the choice of solvents in an attempt to enhance the separation or resolve the tested DHP enantiomers, three most common nonstandard solvents including dichloromethane, tetrahydrofuran, or ethyl acetate were investigated. However, unsuccessful results were achieved using nonstandard mobile phase consisting of hexane with above solvents for the tested enantiomers. We concluded that on Chiralpak IC column at nonstandard elution mode, the high polarity of the solvent might influence the chiral recognition of the column, resulting in poor resolution.

#### 2.1.3. Influence of the Alcohol Modifiers on Enantioseparation

In chromatographic studies, not only the nature of alcohol modifier, but also the concentration of a given mobile-phase modifier, affect the chiral recognition of chiral compounds. Wang et al. [19] have reported that the interactions between the analytes and CSP depended on the nature of the alcohol, and the changes caused on the CSP structure by different alcohols may affect chiral recognition. In order to demonstrate the effect of the applied four alcohol modifiers (IPA, EtOH, NBA, and NPA) on the enantioseparation, the retention of the compounds on the Chiralpak IC column from Tables S1–S4 were compared. Obviously, when branched alcohol, such as IPA, was used as the alcohol modifier, the retention times of all compounds were longer than that of using linear alcohols, which indicated that the steric effect likely contributed to the decreased strengths of the interactions between the mobile phase and the analytes. One example is depicted in Tables S1 and S2 for the separation of M2. When 10% EtOH was replaced by the same percentage of IPA, retention time of the first eluted enantiomer significantly increased from 8.26 to 15.36 min. However, the stronger interactions did not always result in larger resolution and enantioselectivity factor (α) values. In the case of N11, enantioseparation was enhanced by EtOH (k_1_′ = 11.80, R_s_ = 4.02, α = 1.29) instead of IPA (k_1_′ = 12.95, R_s_ = 1.87, α = 1.19). A regular trend for the effect of polar modifier on the enantioselectivity was not observed, which indicated that other types of interactions (hydrogen bonding, π-π stacking interaction, or dipole-dipole interaction), rather than steric hindrance, were responsible for enantiomeric separation.

**Figure 6:**
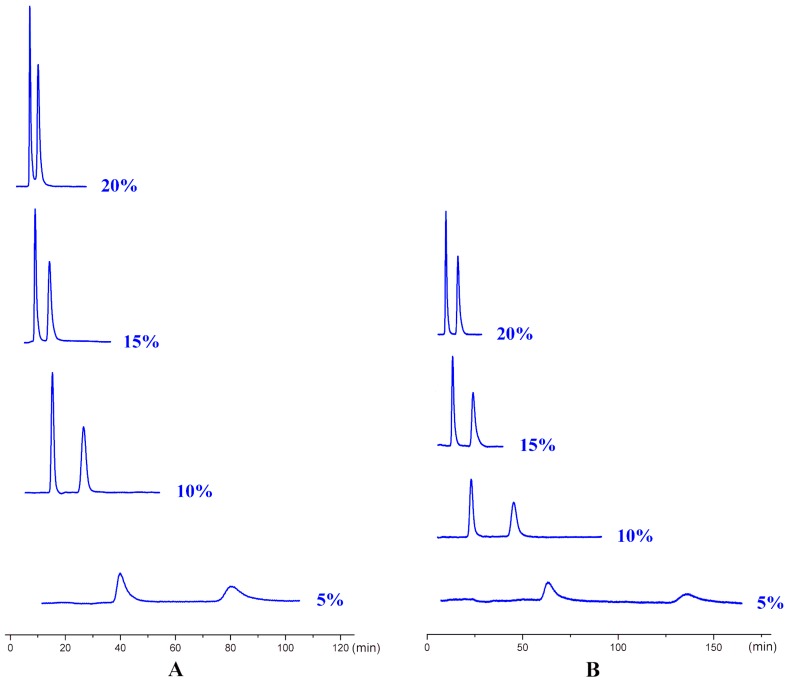
The influence of alcohol content on enantiomeric separation with IPA, (**A**) M2 and (**B**) MD5.

**Figure 7:**
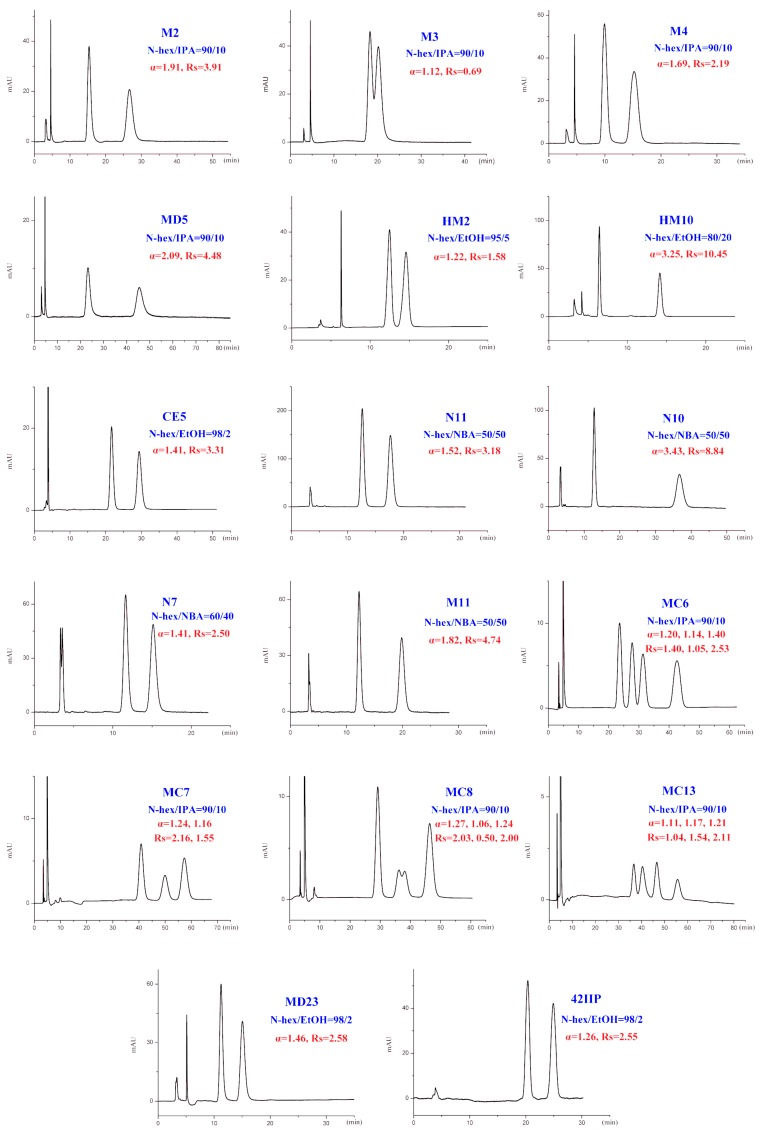
The chromatograms of 17 analytes under optimized conditions of normal-phase mode.

In addition to different polar modifiers, the chiral separation of enantiomers was also dependent on the concentration of modifier adopted. As seen from Tables S1–S4, for M2-4, MD5, MC6-8, and MC13, IPA was more beneficial to their enantiomeric recognition. NBA was in favor of the enantioseparation of N11-10, N7, and M11. Whereas, EtOH exhibited enhanced enantioselectivity to HM2, HM10, CE5, MD23, and 42IIP. Accordingly, the effect of alcohol content on enantioseparation was investigated under these conditions. It was found that in most cases, an increase in the investigated alcohol percentage resulted in a decrease in the retention factors (k’) and enantioselectivity factor (α). For example, increasing the IPA percentage from 5 to 10% produced a notable decrease in k’ values. However, compared to k’ values, a change in the IPA percentage had a smaller effect on α values as only a slight increase in α values was observed with decreasing the IPA percentage. The obtained results suggested that enantiomeric separation depended strongly on the polarity of alcohol modifier. On one hand, by increasing the alcohol content of the mobile phase, the retention was weakened and selectivity of the column was decreased owing to the enhanced competition with analytes for the CSP, appearing in faster elution and lower enantioselectivity factor (α) values. On the other hand, the enantiomeric separation promoted by lower alcohol percentage was always accompanied with prolonged retention times as well as broader peak. Taking M2 and MD5 as example (Figure 6), the peak width obviously broadened when the percentage of the alcohol modifiers in the mobile phase reached to 5%. Therefore, considering of analysis time and resolution, the optimized chromatographic conditions under standard normal mobile phase were as follows: N-hex/IPA (90:10, *v*/*v*) for M2-4, MD5, MC6-8, and MC13, N-hex/EtOH (95:5, *v*/*v*) for HM2, N-hex/EtOH (80:20, *v*/*v*) for HM10. N-hex/NBA (95:5, *v*/*v*) for N11-10 and M11, N-hex/NBA (60:40, *v*/*v*) for N7 and N-hex/EtOH (98:2, *v*/*v*) for CE5, MD23, and 42IIP. The chromatograms of the seventeen analytes using each of the optimized separation condition are shown in Figure 7.

#### 2.1.4. Effects of the Additive

As for any cellulose-derivative-based CSPs, resolution of chiral compounds sometimes requires the combination of an additive into the mobile phase for efficient enantioselective separation of acidic or basic analytes. The role of the additives in the mobile phase is believed to suppress the deleterious effect of residual free silanols on the silica surface and have the acidic or basic solutes eluted in a reasonable range with improved peak symmetry, resolution, and selectivity [20]. For acidic analytes, the common addition is formic acid, while for basic analytes, diethylamine is preferred. Based on that, diethylamine was added to the mobile phase in this study due to the weakly alkalinity of the seventeen chiral DHPs. Under the optimized normal mobile-phase conditions mentioned above, appropriate proportions of diethylamine was applied to evaluate their impacts on the enantioseparation of enantiomers. It was found that the addition of diethylamine to the mobile phase caused a decrease in retention while other relevant improvements in terms of column efficiency were not gained. Even with the addition of diethylamine up to 0.1%, the obvious changes in enantioseparation efficiency including retention factor (k′), resolution, and enantioselectivity factor (α) were not observed, suggesting that the slight changes of basic additives had no influence on the enantioseparation of the tested compounds. After consideration of the destructiveness of basic additives to CSPs, no addition of basic additives was adopted.

### 2.2. Enantioseparation under Reversed-Phase Elution Mode

The cellulose-type CSPs seem to be most frequently used with organic eluents (alkane and polar modifiers). However, enantiomeric separation by aqueous eluent on the cellulose-based CSPs has a history almost as long as their applications with the mobile phases containing alkane mixtures [18]. Apart from the apparent character of the reversed-phase elution for their suitability for liquid chromatography-mass spectrometry (LC-MS) applications, the choice of reversed-phase mode was often related to scarce retentivity of the compounds, the trails with organic mobile phase and the lack of appropriate enantioselectivity in organic eluents. Thus, the feasibility and method development in reversed-phase mode including the mixture methanol (MeOH) or acetonitrile (ACN) with water on Chiralpak IC were conducted. It was found that the resolution of all the analytes decreased with increased percentage of organic solvents (including both MeOH and ACN) in the mobile phases. Faster elution was also observed with increased organic solvents percentage since the increased competition between the organic solvent and analytes. Enantioselectivities with ACN/water were found to be higher than those with MeOH/water. Then, the enantioseparations were optimized by varied percentage of ACN with water or various buffer solution. The typical chromatographic results obtained for the separation of the tested enantiomers on the IC columns are summarized in Table S5.

**Figure 8:**
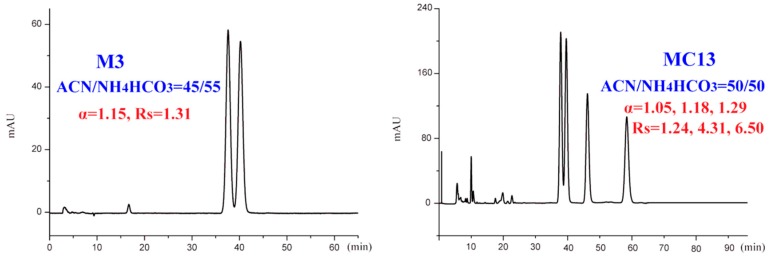
Typical chromatograms with better resolution under optimized conditions of reversed-phase mode. ACN: Acetonitrile.

For the newly synthesized DHP structural analogues, except M2 and M4, all the analytes were obtained enantiomeric separation. In particular, eight out of the seventeen analytes were completely separated, but with longer retention. Compound M3, which was not obtained a baseline separation at the normal-phase elution mode, achieved resolution 1.52 with ACN/20 mM ammonium bicarbonate buffer solution (40:60, *v*/*v*). While for another compound MC13, significantly enhanced enantioseparation was observed at an almost the same longer retention time compared with normal-phase optimized condition. The results indicated that the reversed-phase mode could be sometimes considered as an alternative to offer efficient separation methods while compared with organic eluents. On one hand, increased retention times resulted in an improved enantioresolution. On the other hand, the tailing of chromatographic peaks was suppressed by the presence of buffer salts. As we have seen in the separation of MC13, the resolution was increased while the enantioselectivity, however, kept more or less constant. Therefore, as a comprehensive consideration of resolution, analysis time, and peak shape, the additional mobile-phase conditions of better resolutions were selected and shown in Figure 8.

Based on our extensive experimental results obtained, it became clear that a combination of the different elution mode using Chiralpak IC column could offer a very high success screening of an efficient condition for the variety of racemic DHPs. Such complementariness will be the subject of a comprehensive investigation in the future research of chiral DHPs.

### 2.3. Effects of Temperature and Thermodynamic Parameters

Temperature is certainly of great importance both for optimization of chiral separation and for studying the mechanisms of enantiomeric recognition. In this work, the effect of column temperature on enantioseparation was evaluated with the same chromatographic separation conditions (N-hex/IPA = 90:10) for typical analyte M2, M3, M4, and MD5 which were convenient for discussion. Results and the chromatographic conditions for analytes are given in Table 1.

In general, lower column temperature results in better resolutions, longer retention times and wider peaks, and it was found that the values of retention factor, k′, were decreased with the increasing temperature for all analytes. However, the effect on enantioselectivity factor (α), and resolution (R_s_), varies from case to case. As the column temperature was increased, a corresponding decrease in enantioselectivity factor (α) and resolution (R_s_) values for M2 and M4 were observed, while the values of M3 and MD5 increased. It should be noted that a lower temperature was not always conducive to separation in the first case. The best separation of M2 was obtained at 25 °C with the highest resolution (R_s_) value of 3.91, instead of 20 °C.

Utilizing the chromatographic data, van’t Hoff plots were constructed from the following Equations (1) and (2).

(1) $${\mathrm{lnk}}^{\prime }=-\frac{\Delta H^{{}^{\circ}}}{RT}+\frac{\Delta S^{{}^{\circ}}}{R}+\ln\Phi =-\frac{\Delta H^{{}^{\circ}}}{RT}+\Delta S^{*}$$

(2) $$\ln\alpha =-\frac{\Delta \Delta H^{{}^{\circ}}}{RT}+\frac{\Delta \Delta S^{{}^{\circ}}}{R}$$

where $\Delta H^{{}^{\circ}}$ and $\Delta S^{{}^{\circ}}$ represent the differences in the enthalpy and entropy, respectively, when one enantiomer transfers from the mobile phase to the stationary phase. $\Delta S^{*}$ is used to substitute for the expression $\Delta S^{{}^{\circ}}$/R +lnΦ because of unavailable column phase ratio $\Phi .\;\Delta \Delta H^{{}^{\circ}}$ and $\Delta \Delta S^{{}^{\circ}}$ represent the differences of $\Delta H^{{}^{\circ}}$ and $\Delta S^{{}^{\circ}}$ for a given pair of enantiomers, respectively. *R* is the gas constant. If the plots of lnk′ or lnα against 1/T are linear in a temperature range, the correlative thermodynamic parameters, which are then temperature-independent, can be derived from the slope and the intercept of the straight lines.

The corresponding results were summarized at Table 2. The lnα versus 1/T was highly linear in the temperature range of 20 to 40 °C, indicating that the interaction forces between analytes and the CSP were changed, rather than the change in the conformation of the CSP [21]. As seen from Table 2, the $\Delta H^{{}^{\circ}}$ values of each analyte were quite different, suggesting that the interaction strengths were different between these analytes and the CSP. Additionally, $\Delta \Delta H^{{}^{\circ}}$ and $\Delta \Delta S^{{}^{\circ}}$ values clearly illustrated that the thermodynamic driving forces to chiral separation of all analytes were not the same. Because of the negative $\Delta \Delta H^{{}^{\circ}}$ and $\Delta \Delta S^{{}^{\circ}}$ values, the enantiomeric separation for M2 and M4 were enthalpy-driven. Contrarily, the enantioseparation of the other two analytes were controlled by entropy. Among them, the absolute values of $\Delta \Delta H^{{}^{\circ}}$ of M2 and M4 were larger than others, which showed that interactions related to enantioselectivity between M2 and M4 with CSP were stronger. The absolute value of $\Delta \Delta S^{{}^{\circ}}$ of MD5 was largest, indicating that the degrees of freedom of the second eluted enantiomers was lost the most. By comparison of compounds M2 and M4, the $\Delta H^{{}^{\circ}}$ values of M2 were higher than that of M4, suggesting that the interactions between M4 and the CSP were weak. A possible reason may be that the long alkyl substitution of M4 obstructed this compound from approaching the chiral groove of the CSP, which was consistent with the fact that M2 had the better chiral separation on the column.

As reported in Reference [22], the interaction forces between the analytes and stationary phase could be determined by the absolute value of $\Delta \Delta H^{{}^{\circ}}$. Chiral discrimination was only related to the steric hindrance when the value was less than 0.1 kcal mol^−1^. And as for the value of $\Delta \Delta H^{{}^{\circ}}$ between 0.5 to 1.0 kcal mol^−1^, the contribution of steric hindrance is enlarged by weak interactions such as π-π interactions and hydrogen bonding. When the value is greater than 1.0 kcal mol^−1^, chiral recognition seems to be the result of an additional strong π-π interaction or hydrogen bond for the more retained enantiomer. Hence, from the thermodynamic study view, we could conclude that hydrogen bonding, steric hindrance, and π-π interaction played major roles in the enantiomeric separation of the tested DHP enantiomers.

### 2.4. Application

For application purposes, linearity, limit of quantitation, and repeatability were determined for all studied compounds except M3, MC6-8, and MC13. Method validation was carried out under the optimal chiral separation conditions of normal-phase elution mode. Linear response for the tested analytes was obtained from 0.05–0.30 mg mL^-1^ with a correlation coefficient of greater than 0.9990. The limit of quantitation was estimated to be at least 1 µg mL^−1^ for the compounds. Injection repeatability of six consecutive runs gave relative standard deviations (RSDs) of less than 3%. These results suggest that the present method is effective for the separation and quality control for the newly synthesized structural analogues during their every process.

## 3. Materials and Methods

### 3.1. Chemicals and Reagents

**Figure 9:**
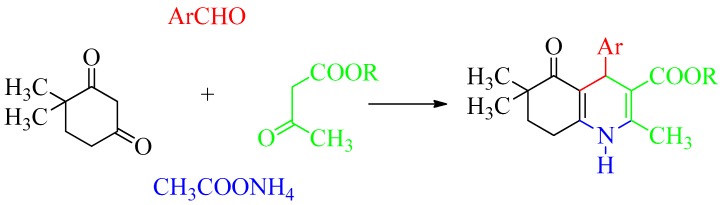
Synthetic scheme for the preparation of newly synthesized DHPs.

The synthesis of the studied compounds M2-4 [4], MD5 [3], HM2 [23], CE5 [24], N7, N10, N11, M11 [25], MC6-8, and MC13 [26] was reported previously. HM10, MD23, and 42IIP were obtained by the reaction of 4,4-dimethyl-1,3-cyclohexanedione, appropriate aromatic aldehyde, and alkyl acetoacetate in ethanol by the presence of excess ammonium acetate (Figure 9). The detailed synthetic procedure and structure-elucidation data for the new compounds are provided as supplementary material (Materials and methods, Figures S1–S6).

N-Hexane (N-hex), isopropanol (IPA), n-propanol (NPA), n-butanol (NBA), ethanol (EtOH), methanol (MeOH), acetonitrile (ACN), tetrahydrofuran, dichloromethane, and ethyl acetate were of HPLC grade and purchased from Concord Tech (Tianjin, China). Analytical grade formic acid, diethylamine, and other components of mobile phase such as ammonium bicarbonate were obtained from Shandong Yuwang Industrial (Shandong, China). All solvents used for mobile phase were passed through a 0.22 μm filter and then degassed in an ultrasonic bath before use.

Sample solutions of the DHPs were prepared by dissolution in the appropriate amounts of MeOH and filtered through a 0.22 µm filter.

### 3.2. Equipment

The Jasco LC-2000 HPLC system (Jasco Corporation, Tokyo, Japan), which consisted of high-pressure binary gradient system, was used for the method development studies, equipped with a PU-2080 pump, an injection-loop of 20 μL, a thermostatted column compartment and a UV-2075 UV-VIS detector. Chromatographic data were acquired and processed by N2000 Chemstation software (Hangzhou, China).

### 3.3. Chromatography

Chiralpak IC column, 250 mm × 4.6 mm I.D. with 5 µm particle size, was obtained from Daicel (Tokyo, Japan). The optimized chromatographic separation conditions were performed at 25 °C with the flow rate of 1 mL min^−1^. The detection was carried out at 230 nm. The hold-up time was measured by the first perturbation of the baseline. Retention or capacity factors, k′, were determined from (t_R_ − t_0_)/t_0_, where t_R_ is the retention time of analyte and t_0_ the hold-up time. Resolution, R_s_, was calculated from 2(t_2_ − t_1_)/(W_1_ + W_2_), where t_1_ and t_2_ are the retention times of the successively eluted enantiomers, and W_1_ and W_2_ are the peak widths of the first and second eluted enantiomers, respectively. Enantioselectivity factor (α) was obtained from k_2_′/k_1_′, where k_1_′ and k_2_′ are the retention factors for the first and the second eluted enantiomer, respectively.

## 4. Conclusions

The solvent versatility of Chiralpak IC column was demonstrated for the enantioselective separation of synthesized DHPs racemic derivatives using different elution modes. The mobile-phase composition, the proportion and nature of the polar modifier, and temperature all affected the chromatographic parameters. Under optimum separation conditions, except four analytes (MC6-8 and MC13) which contained two chiral centers, the tested compounds were all completely separated. It can be seen from the results of different elution modes, standard normal- phase elution mode appeared to be more suitable for the enantioseparation of the investigated compounds. In addition, the use of reversed phase consisting of ACN/ammonium bicarbonate buffer solution broaden the application of the CSP with enhanced resolution comparing to similar separation under standard and nonstandard organic solvents, suggesting that the possibility to use an extended range of solvents in the mobile phase can be a great advantage and lead to enhancement of interactions between the chiral selector and the enantiomers. The possible chiral recognition mechanism was discussed in combination with the enantiomeric separation results and the thermodynamic study. Hydrogen bonding, the steric hindrance, and π-π interaction played major roles in the enantiomeric separation of the tested DHP enantiomers.

## Figures and Tables

**Table 1:** Effects of the column temperature on the retention and enantioseparation.

Analyte	20 °C			25 °C			30 °C			35 °C			40 °C		
	${k^{\prime }}_{1}$	$R_{s}$	α	${k^{\prime }}_{1}$	$R_{s}$	α	${k^{\prime }}_{1}$	$R_{s}$	α	${k^{\prime }}_{1}$	$R_{s}$	α	${k^{\prime }}_{1}$	$R_{s}$	α
**M2**	4.48	3.85	1.95	4.30	3.91	1.91	4.16	3.82	1.88	3.98	3.81	1.84	3.86	3.53	1.81
**M3**	5.54	0.58	1.12	5.31	0.69	1.12	5.06	0.76	1.13	4.82	0.82	1.13	4.65	0.83	1.14
**M4**	2.48	2.15	1.78	2.42	2.14	1.75	2.39	2.11	1.73	2.32	2.02	1.69	2.26	1.94	1.67
**MD5**	7.56	4.43	2.07	7.01	4.48	2.09	6.54	4.57	2.12	5.95	4.66	2.13	5.60	4.68	2.14

Conditions: Mobile phase, N-hex/IPA (90:10, *v*/*v*); flow rate, 1.0 mL min^−1^.

**Table 2:** The thermodynamic parameters for the enantioseparations of studied compounds.

Analyte	$\Delta {H^{{}^{\circ}}}_{1}\;\left(\mathrm{kJ}\;{\mathrm{mol}}^{-1}\right)$	$\Delta S_{1}^{*}\left(J\;K^{-1}{\mathrm{mol}}^{-1}\right)$	$r^{2}$	$\Delta {H^{{}^{\circ}}}_{2}\;\left(\mathrm{kJ}\;{\mathrm{mol}}^{-1}\right)$	$\Delta S_{2}^{*}\left(J\;K^{-1}{\mathrm{mol}}^{-1}\right)$	$r^{2}$	$\Delta \Delta H^{{}^{\circ}}\left(\mathrm{kJ}\;{\mathrm{mol}}^{-1}\right)$	$\Delta \Delta S^{{}^{\circ}}\left(J\;K^{-1}{\mathrm{mol}}^{-1}\right)$
**M2**	−5.74	−0.85	0.9972	−8.54	−1.34	0.9974	−2.80	−4.02
**M3**	−6.81	−1.08	0.9975	−6.19	−0.71	0.9977	0.64	3.11
**M4**	−3.50	−0.52	0.9873	−6.06	−0.99	0.9933	−2.56	−3.91
**MD5**	−11.67	−2.76	0.9970	−10.39	−1.51	0.9952	1.29	10.46

Conditions: Mobile phase, N-hex/IPA (90:10, *v*/*v*); flow rate, 1.0 mL min^−1^.

## Supplementary Materials

Supplementary materials can be found at https://www.mdpi.com/1422-0067/20/10/2513/s1.

## Abbreviations

DHP	Dihydropyridine
CE	Capillary electrophoresis
SFC	Supercritical fluid chromatography
CEC	Capillary electrochromatography
GC	Gas chromatography
HPLC	High-performance liquid chromatography
CSP	Chiral stationary phase
N-hex	N-Hexane
IPA	Isopropanol
NPA	N-propanol
NBA	N-butanol
EtOH	Ethanol
MeOH	Methanol
ACN	Acetonitrile
