# Supplementary material for: Direct Enantiomeric Resolution of Seventeen Racemic 1,4-Dihydropyridine-Based Hexahydroquinoline Derivatives by HPLC
Source: Int J Mol Sci. 2019 May 22;20(10):2513. doi: 10.3390/ijms20102513 (PMC6566779; doi:10.3390/ijms20102513)
Supplement: Supplementary file 1 [file ijms-20-02513-s001.pdf]

Supplementary information

Direct Enantiomeric Resolution of Seventeen Racemic 1,4-Dihydropyridine-Based Hexahydroquinoline Derivatives by HPLC

Jiayi Sun [1], Miyase Gözde Gündüz [2], Junyuan Zhang [1], Jia Yu [1,*], Xingjie Guo [1,*]

The general procedure for the synthesis of DHP derivatives: 1 mmol 1,3-cyclic diketone (4,4-dimethyl-1,3-cyclohexanedione), 1 mmol aromatic aldehyde, 1 mmol appropriate alkyl acetoacetate and excess amount of ammonium acetate were dissolved in absolute ethanol and refluxed for 6 h. After completion of the reaction, monitored by TLC, the reaction mixture was cooled, poured into ice-water. The obtained precipitate was filtered and this crude solid was purified by recrystallization from ethanol-water.

Materials and methods

All chemicals and solvents were purchased from commercial sources (Sigma-Aldrich and Merck) and were used without further purification. Melting points were determined using Thomas Hoover Capillary Melting Point Apparatus (Philadelphia, PA, USA) without calibration. 1H-NMR and 13C-NMR spectra were obtained on a Varian Mercury 400, 400 MHz High Performance Digital FT-NMR Spectrometer (Palo Alto, CA, USA) in dimethylsulfoxide (DMSO-d6) solutions. The ESI-MS spectra were carried out on a micro mass ZQ-4000 single quadrupole mass spectrometer (Waters, Eichhorn, Germany).

2-(Methacryloyloxy)ethyl 4-(3,5-dichloro-2-hydroxyphenyl)-2,6,6-trimethyl-5-oxo-1,4,5,6,7,8-hexahydroquinoline-3-carboxylate (HM10): Prepared by the reaction of 4,4-dimethyl-1,3-cyclohexanedione, 3,5-dichlorosalicylaldehyde, 2-(methacryloyloxy)ethyl acetoacetate and ammonium acetate. Yellowish solid, yield: 62%. m.p. 171-173. ^{1}H-NMR (δ, DMSO-*d*$_6$): 0.95 (3H; s; 6-CH_3), 1.02 (3H; s; 6-CH_3), 1.65-1.71 (2H; m; H-7), 1.79 (3H; s; -$C(C\underline{H_3})=CH_2$), 2.37 (3H, s, 2-CH_3), 2.51-2.55 (2H; m; H-8), 4.06-4.22 (4H; m; -$COOC\underline{H}_2C\underline{H}_2OCO$-), 4.86 (1H; s; H-4), 5.61 (1H; s; -$C=CH_{2A}$), 5.87 (1H; s; -$C=CH_{2B}$), 6.88 (1H, d, J=2.4 Hz, Ar-H_6), 7.19 (1H, d, J=2.4 Hz, Ar-H_4), 9.64 (1H; s; N-H), 10.45 (1H; s; O-H). ^{13}C-NMR (δ, DMSO-d$_6$): 17.7 ($C(\underline{C}H_3)=CH_2$), 18.1 (2-CH_3), 23.2 (6-CH_3), 24.1 (6-CH_3), 25.1 (C-8), 31.2 (C-7), 33.2 (C-4), 39.5 (C-6), 61.2 (-$O\underline{C}H_2CH_2O$-), 62.4 (-$OCH_2\underline{C}H_2O$-), 102.6 (C-3), 107.8 (C-4a), 122.2, 123.1, 125.7, 127.0 (phenyl carbons) 126.4(-$C=\underline{C}H_2$), 135.3 (-$\underline{C}(CH_3)=CH_2$), 137.5, 146.6 (phenyl carbons), 148.4 (C-2), 153.7 (C-8a), 165.9 (-$\underline{C}OOCH_2$-), 166.1(-$\underline{C}OC(CH_3)=CH_2$), 203.4 (C-5).ESI-MS(m/z):530/532/534 $[M+Na]^+/[M+2+Na]^+/[M+4+Na]^+$.

Ethyl 4-(2,5-bis(trifluoromethyl)phenyl)-2,6,6-trimethyl-5-oxo-1,4,5,6,7,8-hexahydroquinoline-3-carboxylate (MD23): Prepared by the reaction of 4,4-dimethyl-1,3-cyclohexanedione, 2,5-bis(trifluoromethyl)benzaldehyde, ethyl acetoacetate and ammonium acetate. Yellowish solid, yield: 58%. m.p. 193-195. ^{1}H-NMR (δ, DMSO-*d*$_6$): 0.77 (3H; t; *J*= 7.2 Hz, $CH_2C\underline{H}_3$), 0.86 (3H; s; 6-CH_3), 0.95 (3H; s; 6-CH_3), 1.67-1.76 (2H; m; H-7), 2.27 (3H; s; 2-CH_3), 2.47-2.52 (2H; m; H-8), 3.65-3.77 (2H; m; $C\underline{H}_2CH_3$), 4.78 (1H; s; H-4), 6.82 (1H; d; *J*= 8.8 Hz; Ar-H_3), 7.14 (1H; dd; *J*= 8.8 / 2.0 Hz; Ar-H_4) ; 7.23 (1H; d; *J*= 2.0 Hz; Ar-H_6), 9.07 (1H; s; N-H). ^{13}C-NMR (δ, DMSO-*d*$_6$): 13.8 ($CH_2\underline{C}H_3$), 18.2 (2-CH_3), 22.9 (6-CH_3), 24.1 (6-CH_3), 24.3 (C-8), 33.8 (C-7), 34.1 (C-4), 39.2 (C-6), 59.0 ($\underline{C}H_2CH_3$), 103.4 (C-3), 109.6 (C-4a), 122.1 (phenyl carbon), 123.0 (CF_3), 124.8, 125.7 (phenyl carbons), 127.1 (CF_3), 129.7, 132.2, 144.9 (phenyl carbons), 148.7 (C-2), 150.0 (C-8a), 166.5 (-$\underline{C}OOCH_2$-), 199.0 (C-5). ESI-MS (m/z): 498 $[M+Na]^+$

Isopropyl 4-(1-methyl-1*H*-indol-2-yl)-2,6,6-trimethyl-5-oxo-1,4,5,6,7,8-hexahydroquinoline-3-carboxylate (42IIP): Prepared by the reaction of 4,4-dimethyl-1,3-cyclohexanedione, 1-methylindole-2-carboxaldehyde, isopropyl acetoacetate and ammonium acetate. Yellow crystal, yield: 38%. m.p. 240-242. ^{1}H-NMR (δ, DMSO-d_6): 0.84 (3H; s; 6-CH_3), 0.91 (3H; d; *J*=6.0 Hz, -CH-(C$\underline{H}_3$)$_2$), 0.97 (3H; s; 6-CH_3), 1.13 (3H; d; *J*=6.0 Hz, -CH-(C$\underline{H}_3$)$_2$), 1.64-1.70 (2H; m; H-7), 2.29 (3H, s, 2-CH_3), 2.45-2.55 (2H; m; H-8), 3.39 (3H; s; N-CH_3), 4.74-4.81 (1H; m; -C$\underline{H}$-(CH_3)$_2$), 4.88 (1H; s; H-4), 5.98 (1H; s; indole-H_3), 6.87, 6.98 (4H; t; *J*=8.4 Hz; indole-$H_{5,6}$), 7.31 (2H; d; *J*=8.4 Hz; indole-$H_{4,7}$), 9.18 (1H; s; N-H). ^{13}C-NMR (δ, DMSO-d6): 18.5 (2-CH_3), 21.6, 21.7 (-CH($\underline{C}H_3$)$_2$), 23.0 (6-CH_3), 24.1 (6-CH_3), 25.0 (C-8), 28.3 (N-CH_3), 29.6 (C-7), 33.9 (C-6), 39.5 (C-4), 66.1 (-COO$\underline{C}$H(CH_3)$_2$), 98.7 (indole carbon), 103.4 (C-3), 109.2 (C-4a), 109.4, 118.5, 119.1, 119.8, 127.4, 135.9, 144.1 (indole carbons), 148.9 (C-2), 149.0 (C-8a), 166.4 (-$\underline{C}$OOCH-),199.7 (C-5). ESI-MS (m/z): 429 [M+Na]$^+$

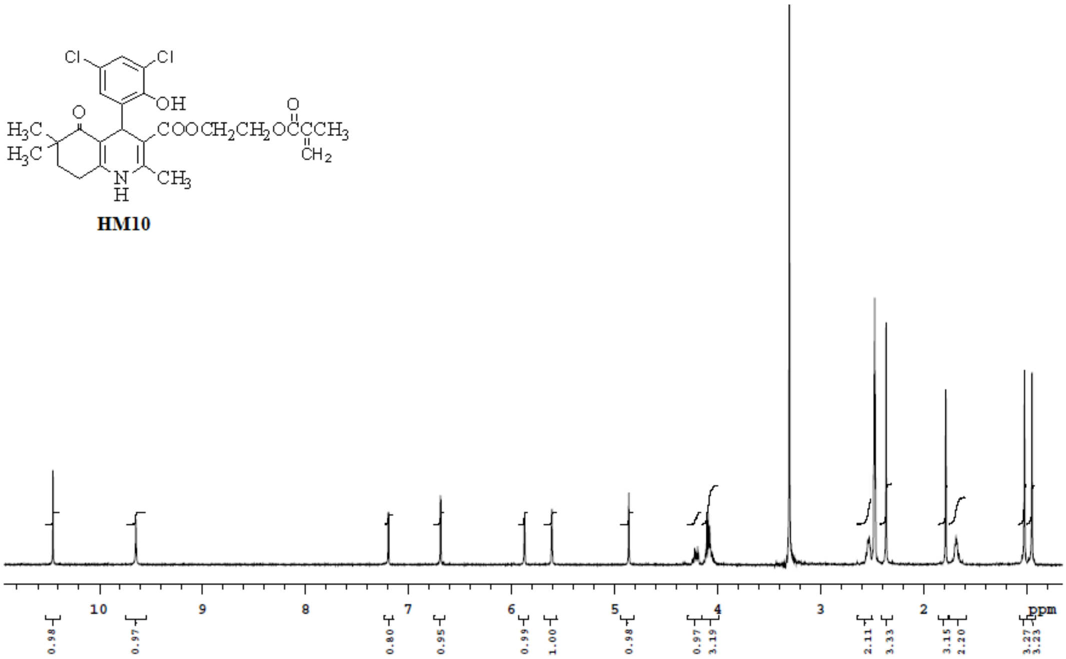

Figure S1. ^{1}H-NMR spectrum of HM10

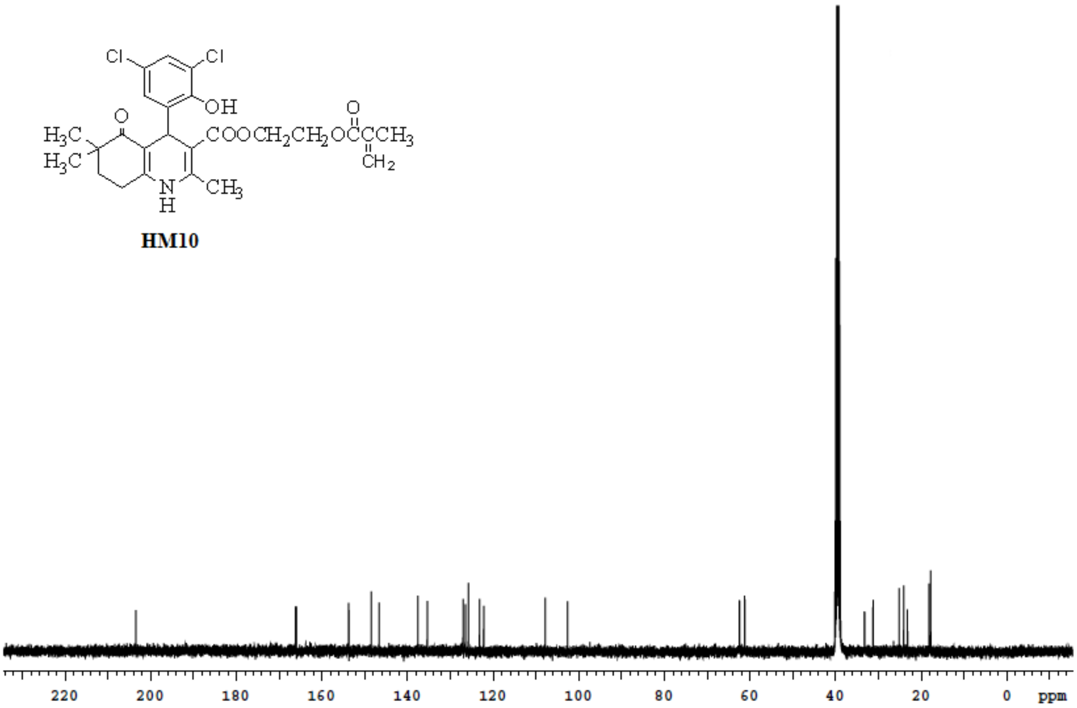

Figure S2. ^{13}**C-NMR spectrum of HM10**

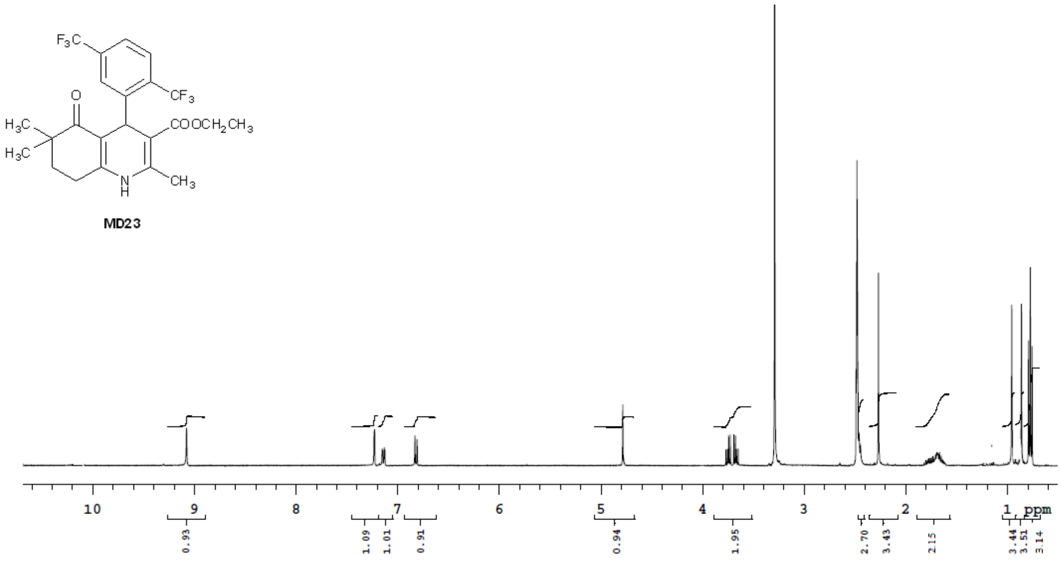

Figure S3. ^{1}**H-NMR spectrum of MD23**

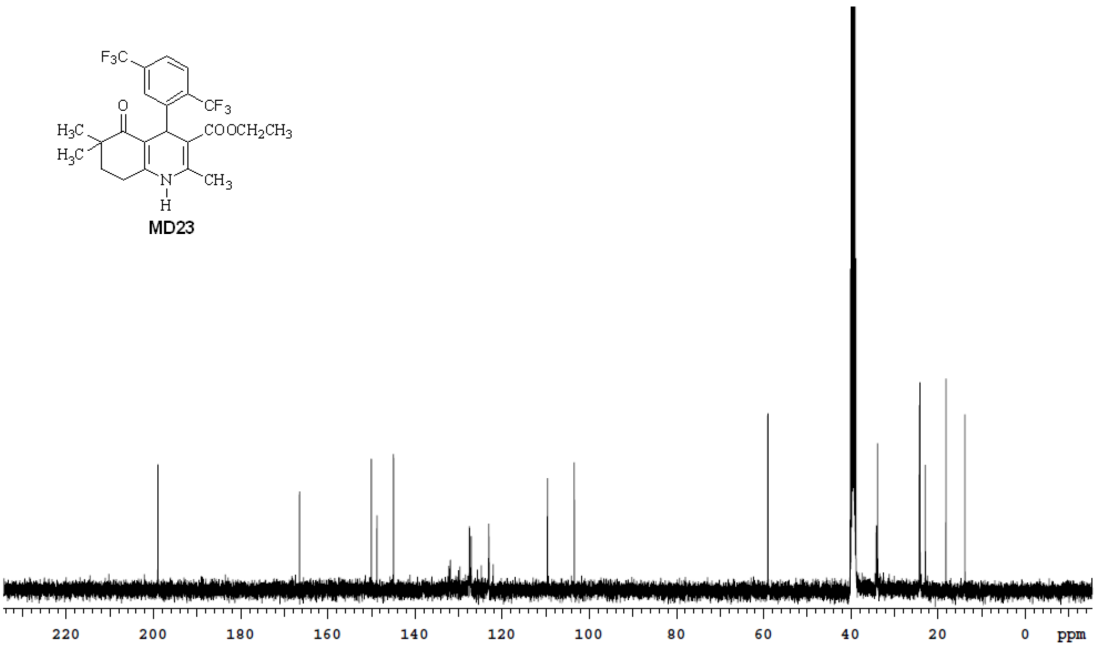

Figure S4. ^{13}C-NMR spectrum of MD23

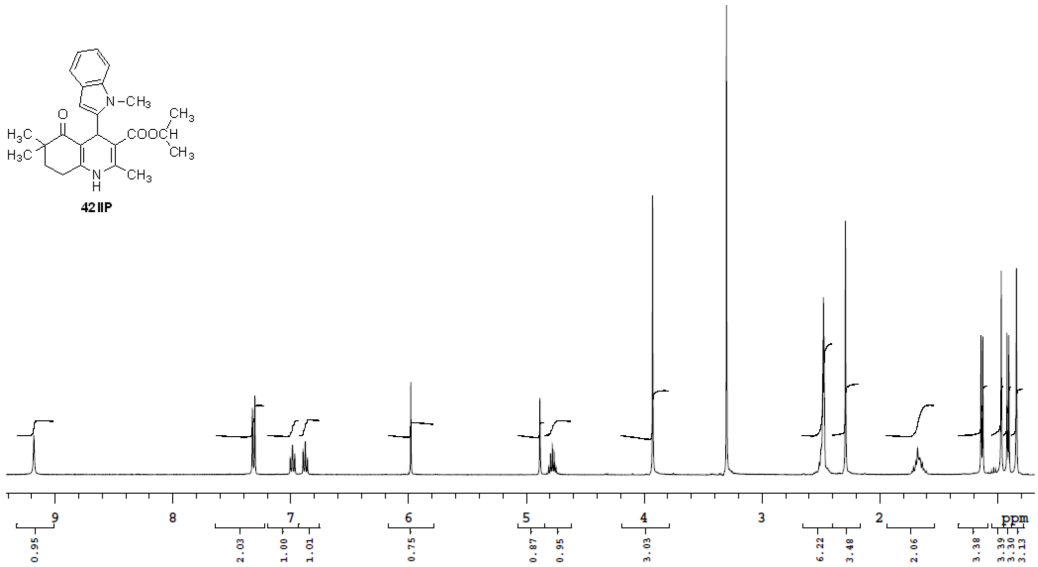

Figure S5. ^{1}H-NMR spectrum of 42IIP

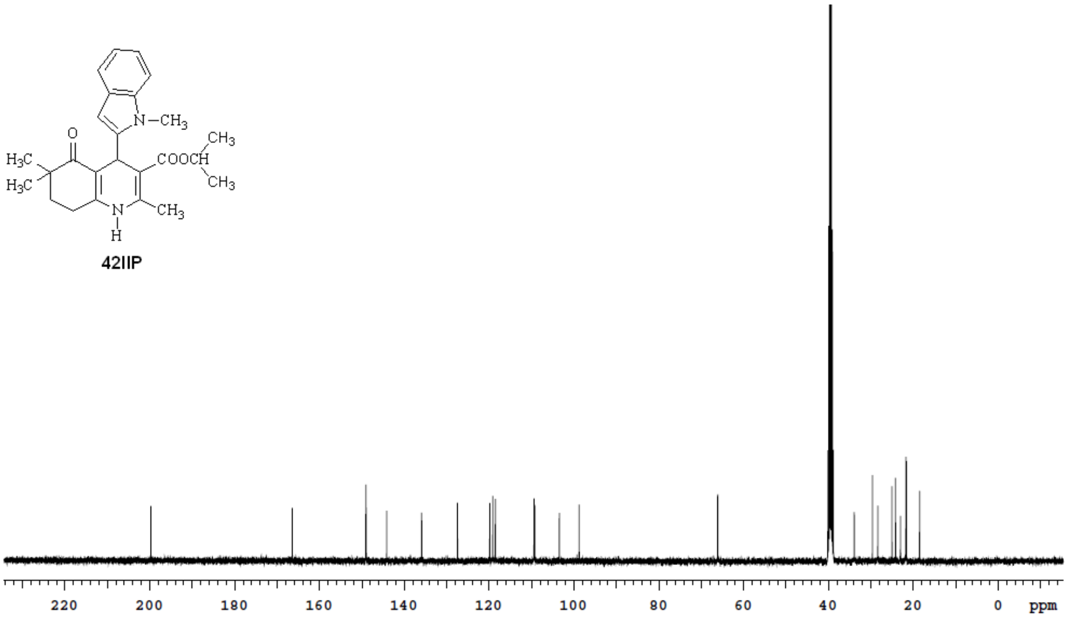

Figure S6. ^{13}**C-NMR spectrum of 42IIP**

Table S1. Effects of the content of IPA in the mobile phase on the retention and enantioseparation.

Analyte	Alcohol content (%)	k'_1	R_s	α
M2	20	1.42	2.19	1.73
	15	2.26	2.75	1.79
	10	4.30	3.91	1.91
M3	20	1.84	0	1.00
	15	2.99	0	1.00
	10	5.31	0.69	1.12
M4	20	0.83	1.16	1.64
	15	1.31	1.52	1.67
	10	2.42	2.14	1.75
MD5	20	2.40	3.02	1.91
	15	3.71	3.57	2.00
	10	7.01	4.48	2.09
	5	20.88	5.77	2.21
HM2	20	1.75	0.59	1.23
	10	3.84	0.92	1.26
	5	11.34	1.63	1.34

HM10	25	2.47	16.38	7.81
CE5	10	1.26	1.09	2.23
	5	6.20	1.69	2.27
N11	30	12.95	1.87	1.19
	25	21.81	2.44	1.21
N10	30	13.29	11.76	3.11
N7	30	6.22	3.31	1.41
	20	26.51	4.94	1.54
M11	30	14.02	4.65	1.51
MC6*	15	3.40/4.08/4.44	1.12/0.53/1.92	1.20/1.09/1.35
	10	6.87/8.26/9.45	1.40/1.05/2.53	1.20/1.14/1.40
MC7*	15	5.80/7.18	1.62/0.87	1.24/1.11
	10	12.63/15.65	2.16/1.55	1.24/1.16
MC8*	15	4.54/5.88	1.62/1.44	1.29/1.23
	10	8.75/11.08/11.72	2.03/0.50/2.00	1.27/1.06/1.24
MC13*	15	6.35/8.01	1.74/1.26	1.26/1.14
	10	11.23/12.48/14.54	1.04/1.54/2.11	1.11/1.17/1.21

* MC6, MC7, MC8 and MC13 molecules possessed four enantiomers, respectively. Conditions: flow rate, 1.0 mL min^{-1}; column temperature, 25 ℃.

Table S2. Effects of the content of EtOH in the mobile phase on the retention and enantioseparation.

Analyte	Alcohol content (%)	k'_1	R_s	α
M2	15	1.10	0.53	1.13
	10	1.85	0.76	1.16
	5	4.61	1.36	1.22
M3	15	1.24	0	1.00
	10	2.09	0	1.00
	5	5.29	0	1.00
M4	15	0.79	0	1.00
	10	1.16	0.49	1.12
	5	2.67	0.99	1.19

MD5	15	1.28	1.61	1.48
	10	2.15	2.23	1.55
	5	5.59	3.24	1.69
HM2	10	1.85	0.99	1.19
	5	3.16	1.58	1.22
HM10	20	1.14	10.45	3.25
	15	1.74	12.16	3.40
	10	5.11	15.71	3.72
CE5	5	1.84	1.10	1.25
	2	6.22	3.31	1.41
N11	20	6.67	3.38	1.27
N10	20	6.90	9.88	2.02
N7	20	3.80	0.75	1.07
M11	20	8.04	3.86	1.30
MD23	2	2.75	2.58	1.46
42IIP	5	1.53	0.88	1.14
	2	5.79	2.55	1.26

Conditions: flow rate, 1.0 mL min^{-1}; column temperature, 25 ℃.

Table S3. Effects of the content of NPA in the mobile phase on the retention and enantioseparation.

Analyte	Alcohol content (%)	k'_1	R_s	α
M2	20	0.80	0.98	1.34
	10	2.11	2.23	1.42
	5	5.67	3.18	1.52
M3	20	0.92	0	1.00
	10	2.47	0	1.00
	5	6.57	0.50	1.07
M4	20	0.52	0.48	1.20
	10	1.26	1.35	1.32
	5	3.14	2.20	1.42
MD5	20	1.07	1.86	1.62

	10	2.91	3.43	1.76
	5	8.15	4.32	1.91
N11	45	2.38	2.29	1.32
	30	5.23	3.17	1.37
N10	50	2.03	6.70	2.43
	30	5.26	9.76	2.80
N7	30	3.10	0.48	1.06
	20	7.28	0.70	1.07
M11	45	2.58	3.30	1.47
	30	5.62	4.46	1.54
MC8*	5	14.20/16.27/16.58	1.73/0.25/1.59	1.15/1.02/1.12
MC13*	5	16.88/19.45/19.67	2.85/0.22/2.29	1.15/1.01/1.15

* MC8 and MC13 molecules possessed four enantiomers, respectively. Conditions: flow rate, 1.0 mL min^{-1}; column temperature, 25 ℃.

Table S4. Effects of the content of NBA in the mobile phase on the retention and enantioseparation.

Analyte	Alcohol content (%)	k'_1	R_s	α
M2	20	1.02	1.75	1.51
	10	2.59	2.93	1.65
	5	6.92	3.97	1.81
M3	20	1.26	0	1.00
	10	3.25	0	1.00
	5	8.57	1.05	1.17
M4	20	0.64	0.91	1.34
	10	1.52	1.89	1.48
	5	3.81	2.75	1.64
MD5	20	1.54	1.88	1.55
	10	4.00	2.96	1.73
	5	10.85	4.03	1.94
HM2	20	1.05	1.12	1.35
	10	2.74	1.63	1.36

	5	6.97	1.97	1.37
HM10	50	0.61	9.50	6.47
	40	0.83	12.37	7.45
	20	2.75	15.34	7.90
N11	50	3.21	3.18	1.52
	40	5.48	3.83	1.59
	30	10.20	4.68	1.63
N10	50	3.27	8.84	3.43
N7	50	1.79	1.96	1.33
	40	2.88	2.50	1.41
	20	13.10	4.16	1.49
M11	50	3.09	4.74	1.82
	40	5.17	5.49	1.91
	30	9.58	6.73	1.97

Conditions: flow rate, 1.0 mL min^{-1}; column temperature, 25ºC.

Table S5. The results for test compounds under reversed phase mode.

**Analyte**	**ACN-20mM ammonium bicarbonate (%)**	$\mathbf{k'_1}$	$\mathbf{R_s}$	$\boldsymbol{\alpha}$
M2	40:60	9.12	0	1.00
M3	45:55	12.38	1.31	1.15
	40:60	22.68	1.52	1.08
M4	40:60	9.49	0	1.00
MD5	45:55	5.32	1.57	1.12
HM2	50:50	12.07	2.29	1.09
HM10	50:50	10.27	7.05	1.32
CE5	50:50	5.09	0.75	1.04
N11	50:50	6.23	2.90	1.14
N10	50:50	12.22	11.49	1.69
N7	50:50	6.94	2.15	1.10
M11	50:50	4.65	2.77	1.14
MC6	50:50	5.93	2.93	1.13
MC7	40:60	8.91/9.92	2.00/0.84	1.11/1.05

MC8	40:60	11.18/12.38	2.00/1.43	1.11/1.08
MC13	50:50	11.84/12.44/14.73	1.24/4.31/6.50	1.05/1.18/1.29
MD23	50:50	3.79	0.71	1.03
42IIP	50:50	4.60	1.03	1.04

Conditions: flow rate, 1.0 mL min-1; column temperature, 25 ℃.
